# Hydroxychloroquine-Induced Stevens–Johnson Syndrome in the Patient with Systemic Lupus Erythematosus: A Case Report in Kurdish Region - Iraq

**DOI:** 10.31138/mjr.260723.his

**Published:** 2023-07-26

**Authors:** Niaz Al-Barzinji, Aryan Mohamadfatih Jalal

**Affiliations:** 1 College Of Medicine, Hawler Medical University, Erbil, Iraq,; 2 Department of Rheumatology, Kurdistan Board for Medical Specialties, Erbil, Iraq

**Keywords:** systemic lupus erythematosus, hydroxychloroquine, Stevens-Johnson Syndrome

## Abstract

Systemic lupus erythematosus (SLE) is a chronic autoimmune disease that mainly affects women. Hydroxychloroquine (HCQ) and chloroquine are widely used in the treatment of many diseases, such as malaria, rheumatic arthritis, systemic lupus erythematosus, and other Rheumatic diseases. The Stevens-Johnson Syndrome (SJS) is a rare immune complex-mediated hypersensitivity disorder that is characterised as a vesiculobullous erythema multiform of the skin, mouth, eyes, and genitals. We decided to report a thirty-year-old female patient with HCQ-developed side effects of induced SJS and its appropriate management. In conclusion, the HCQ tablet does have known side effects. One of the side effects of HCQ is SJS caused by the drug; given the worldwide use of this drug in Rheumatic diseases and its increasing need, we need to be careful about its use to control and manage its side effects.

## INTRODUCTION

Systemic lupus erythematosus (SLE) is one of the chronic autoimmune diseases that mainly affects women. The disease has a relapsing and remitting course. Many organs and tissues are involved, such as, the heart, the kidneys, and the brain as well as the skin, the joints, the pericardium, and the pleura.^1^

Stevens-Johnson Syndrome (SJS) is a rare immune complex-mediated hypersensitivity disorder that is described as a vesiculobullous erythema multiforme of the skin, eyes, mouth, and genitals.^2^ Medications seem to be the most common cause of SJS and have been implicated in as many as 60% of cases studied.^3^

SJS and toxic epidermal necrolysis (TEN) are severe cutaneous adverse reactions (SCARs), carrying an associated mortality from 5–40%. Known risk factors for SJS/TEN include female gender and certain HLA genotypes.^4,5^ Various medications have been described to cause SJS/TEN with strongest associations with, antiepileptics, allopurinol, sulfa-containing antibiotics, non-steroidal anti-inflammatory agents (NSAIDs), β-lactam antibiotics, quinolones, and nevirapine.^6,7^

HCQ and chloroquine are widely used in the treatment of many diseases such as malaria, systemic lupus erythematosus, rheumatic arthritis, and other Rheumatic diseases.

We decided to report a thirty-year-old female patient with HCQ-developed side effects of induced SJS and its appropriate management.

## CASE REPORT

A thirty-year-old female, a known patient of SLE since 1 year (July 2021) which was diagnosed based on history of joint pain of (hands, wrist, knee joints) without swelling, morning stiffness of 30 minute duration, with hair loss, photosensitivity, and oral ulcerations, her Anti-nuclear antibody (ANA) were positive diagnosed according to 2019 ACR/EULAR criteria for SLE, family history positive for rheumatic diseases (mother had systemic sclerosis for 30 years and on treatment, sister has been diagnosed with SLE since 3 years and on treatment). The patient at that time (July 2021) started on nonsteroidal anti-inflammatory (NSAID) with disease-modifying antirheumatic drugs (DMARDs; Methotrexate ampule 15 mg), and folic acid. She took treatment for two months, to which she responded very well. She stopped the treatment by herself, and she was free from any complaints, until on 27 June 2022 she visited the department of Rheumatology with recurrence of symptoms of joint pain with oral ulceration and photosensitivity.

After blood investigations her ESR was 40, complete blood count showed Haemoglobin 10, platelets 150, WBC 4. General urine examination no protein, no cast, C3, C4 normal, so patient started on Hydroxychloroquine tablet 200 mg BID. After taking the Hydroxychloroquine on 8^th^ of July 2022 she started to develop severe headache, with vomiting without blood, with skin rash-like redness, small rash at lower limb, then involving upper limb after 2 days, then involving back, trunk, and face. She visited hospital diagnosed as measles and was prescribed topical treatment. She exacerbated the rash which was painful, with vesicle with pus discharge and burn-like. Her eye also involved difficulty to open eyes, no cough, no dyspnoea, constipation, continuous high-grade fever. She then visited the Rheumatology department; the patient was then admitted to hospital ICU. On examination, patient was conscious and not dyspnoeic, no jaundice, with eruptive skin rash of macule papule vesicle with desquamation and burn-like rash overlying the whole face, pruritic erythematous maculopapular rash and flat atypical targets that started from the distal of upper extremities (rapidly involved the entire body and torn blisters upper limb, lower limb, trunk, back, with oral, genital, and gluteal region involved). Her vital signs were: pulse rate 100 beats/minute; temperature 38.9; SPO2 98 on room air; BP 100/60; cardiovascular and respiratory examination were unremarkable. musculoskeletal examination. Her investigations showed CBC (WBC 4.1, PLT: 165, HGB: 11.7), CRP 55.87mg/L (normal range <5.0), D-dimer 2849.23 (<400), Albumin 3.89g/dl (3.40–4.80), Alkaline phosphates 164.1U/L (70.0–380.0), ALT14.3 u/l (7.0–35.0, AST 31.3 U/L (13.0–31.0), Creatinine 0.90mg/dl (0.2–1.1), urea8 mg/dl (13–43), phosphorus 2.52 mg/dl (2.8–4.1), viral Hepatitis B, C, and HIV negative. Finally, a diagnosis of SJS was made. HCQ was discontinued, then patient started on treatment by applying canula, and foley catheter (fluid replacement G/S+N/S 1*4 daily, with Ceftriaxone vial 1g BID, par acetol bottle 1*3, dexamethasone 8mg BID, Heparin 1cc BID, lactulose syrup for constipation, artificial eye drops for eye, Tel fast tab), with daily follow-up of patient with team consist of internist, Rheumatologist, and plastic. After 10 days remaining in hospital, she became better clinically and by investigation, and she discharged after three months she recovered fully, with only some sort of skin exfoliation remaining on distal forearm.

**Figure 1.**
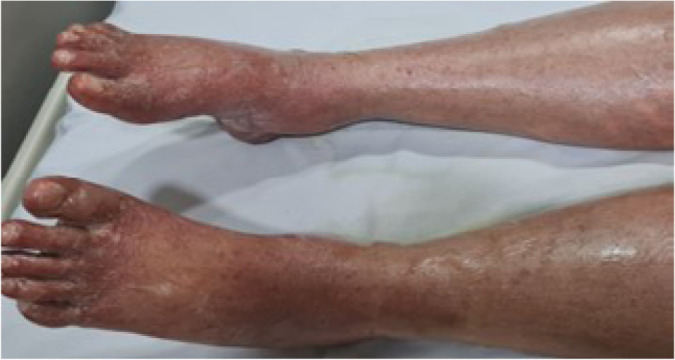
Multiple erythematous exfoliated patches on an erythematous base on the arm.

**Figure 2.**
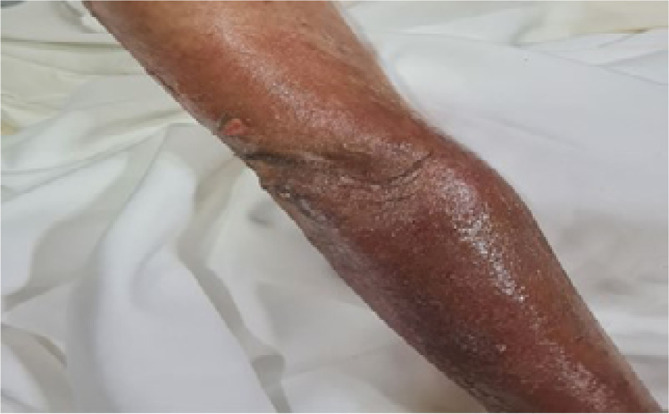
Erythematous exfoliated patches with multiple erosion on erythematous base on the distal extremities of lower limb.

## DISCUSSION

Chloroquine and its derivatives, such as HCQ, have a long history of being used as prophylactic drugs in areas plagued by malaria.^8^ In a study conducted by Davoudi L et al.,^9^ acute generalised exanthemata’s pustulosis, SJS, toxic epidermal necrolysis and rashes. HCQ is a very rare cause of drug-induced SJS. This condition begins as itchy popular erythematous eruptions in the trunk and then affects the face, organs, and mucous membranes of the mouth. It also had purulent rashes on the trunk, limbs, and face.^10^ In a study by Volpe et al.11 was a patient of rheumatoid arthritis after taking HCQ developed diffuse, erythematous exfoliative rash involving trunk and limbs, which diagnosed as a drug rash with eosinophilia and systemic symptoms syndrome. In another study, after taking HCQ, the patient developed skin symptoms of SJS such as a pruritic rash over her abdomen, which described as ‘targets’ with a persisting exfoliating rash and eczematous patches.^12^

Numerous studies have shown that the use of HCQ in some patients with COVID-19 may cause gastrointestinal symptoms and heart problems,^13–15^ but so far there have been no reports that taking HCQ leads to the symptoms of SJS, which develops skin rashes in different parts of the body, especially in the oropharynx. Skin manifestations of COVID-19 included erythematous rash, urticaria, and chickenpox-like vesicles.^16^

## CONCLUSIONS

The HCQ tablet does have known side effects. One of the side effects of HCQ is SJS caused by the drug, and given the worldwide use of this drug in Rheumatic diseases and the increasing need for this drug, we need to be careful about its use to control and manage its side effects.
